# Biomarker-Defined Subsets of Common Diseases: Policy and Economic Implications of Orphan Drug Act Coverage

**DOI:** 10.1371/journal.pmed.1002190

**Published:** 2017-01-03

**Authors:** Aaron S. Kesselheim, Carolyn L. Treasure, Steven Joffe

**Affiliations:** 1 Program On Regulation, Therapeutics, And Law (PORTAL), Division of Pharmacoepidemiology and Pharmacoeconomics, Department of Medicine, Brigham and Women’s Hospital and Harvard Medical School, Boston, Massachusetts, United States of America; 2 Department of Medical Ethics & Health Policy, University of Pennsylvania Perelman School of Medicine, Philadelphia, Pennsylvania, United States of America; 3 Leonard Davis Institute of Health Economics, University of Pennsylvania, Philadelphia, Pennsylvania, United States of America

## Abstract

Aaron Kesselheim and colleagues examine orphan-designated drugs approved between 2009 and 2015 in the United States.

**Summary Points**

- The Orphan Drug Act of 1983 was intended to incentivize the development of pharmaceutical products for rare diseases by providing manufacturers with the opportunity to earn grants, tax credits, fee waivers, and seven years of post-approval market exclusivity for the approved indication.
- Over the past decade, the number of orphan drug designations has roughly doubled, with a simultaneous increase in those that target biomarker-defined subsets of common diseases.
- Among all orphan-designated drugs approved in 2009–2015 indicated for biomarker-defined disease subsets, we examined the circumstances surrounding the drug’s discovery and development, secondary approvals, off-label uses, subsequent revenues, and the reported monthly cost.
- Orphan-designated drugs to treat biomarker-defined subsets of common conditions have a number of characteristics that make them ill-suited to the orphan drug designation, including short development times and rapid expansion of off-label indications after approval. Application of the Orphan Drug Act in these cases risks wasting resources that might be better focused on truly rare conditions.

Congress passed the Orphan Drug Act in 1983 to incentivize the development of pharmaceutical products for rare diseases that might not otherwise be financially viable because of small potential patient populations [1]. Companies can apply for an orphan drug designation from the Food and Drug Administration (FDA) based on the rarity of the targeted disease—defined by a prevalence of fewer than 200,000 patients annually in the United States—and providing a medically plausible basis for believing that their drug or biologic product would aid in its treatment, prevention, or diagnosis. The product is then subject to safety and efficacy testing and formal FDA review and approval.

Orphan drug designation provides manufacturers with the opportunity to earn special research grants from a pool of over US $20 million per year, and subsequent FDA approval of the product carries additional incentives: companies receive tax credits for incurred clinical trial costs (50% tax credit for expenses incurred during clinical testing, maximum of US $30 million), waiver of the FDA approval user fee (currently approximately US $2.4 million), and seven years of post-approval market exclusivity for the approved indication [2]. This legislation has been largely considered a success, with proponents arguing that it has contributed to the commercialization of many drugs in the past 30 years [3]. The number of orphan drug designations has increased from an average of 63 per year in the first two decades of the legislation (1984–2003) to over 200 per year in the past decade (Fig 1). In 2015 alone, 353 products received Orphan Drug Act designations at various stages in their pre-FDA-approval testing process.

**Fig 1:**
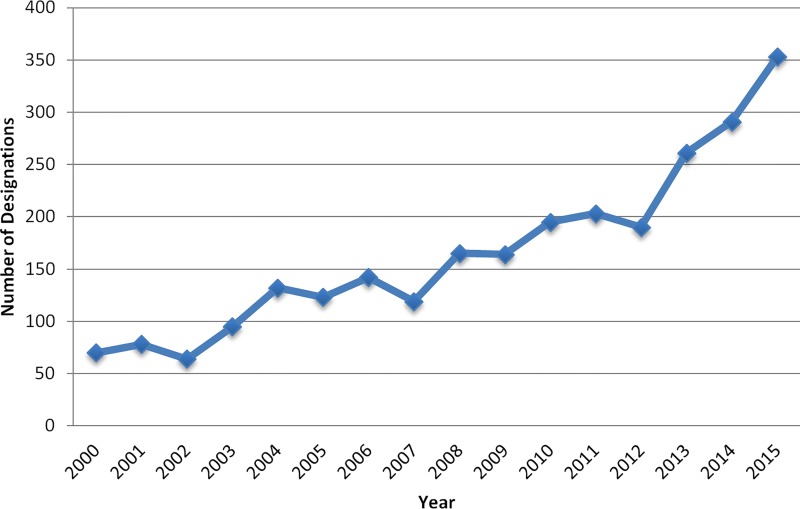
Orphan drug designations per year.

## Yearly Numbers of Drug Products as Qualifying for Orphan Drug Status by the FDA (2000–2015)

Recently, the landscape surrounding use of this act has begun to change. Over the past decade, as orphan drug approvals have comprised an increasing share of all FDA-approved drugs, one contributor to this rise has been an increase in orphan-designated drugs that target biomarker-defined disease subsets [4]. For example, while non-small cell lung cancer was once divided into squamous cell carcinoma and adenocarcinoma, scientists now consider it a heterogeneous disease made up of numerous different genetic aberrancies. About 5% of non-small cell lung cancers have been found to have a rearrangement in the *ALK* gene, and three targeted chemotherapy agents—crizotinib (Xalkori), ceritinib (Zykadia), and alectinib (Alecensa)—have been approved for patients with lung cancer demonstrating *ALK* mutations. All were designated as orphan drugs [5].

With increasing investment in precision medicine, biomarker-defined disease subsets will play an increasingly central role in drug development. We sought to determine to what extent drugs targeting biomarker-defined subsets of more common diseases have been classified as orphan drugs over the past decade. Because the intent of the Orphan Drug Act was to help incentivize for-profit pharmaceutical manufacturers to invest in drugs important for patients with rare diseases, such a shift may signal the need for changes to the legislation.

## Analysis of Orphan-Designated Drugs (2009–2015)

### Data Sources and Collection

Using the FDA’s database of approved drugs, we compiled a list of all therapeutic drugs approved with formal orphan designation from 2009 to 2015 (excluding products used in diagnosis, like contrast agents). We then determined the drug’s primary therapeutic area and whether the orphan-designated drug targeted a biomarker-defined rare subset of a disease. A biomarker-defined subset was specified for this purpose as any drug approved based on its efficacy in a subset of a more prevalent disease characterized by a particular genetic variant or other specified diagnostic test.

### Data Extraction

For each drug, we examined the circumstances surrounding the drug’s discovery and development. We collected certain key characteristics of the pivotal trials leading to the approval of the drugs. A pivotal trial is a clinical trial labeled by the FDA as most important in providing support for the indication(s) for which a drug is approved. From the FDA medical reviews and the published pivotal trials, we identified the total number of participants exposed to the drug and whether the trial tested a surrogate endpoint (e.g., disease response, disease progression) or clinical endpoint (e.g., overall survival). We also identified the date of the Investigational New Drug application, signaling the initiation of human clinical trials, and the date of New Drug Application to determine the length of time each drug spent in active development.

Next, we identified other special FDA designations—priority review status, accelerated approval, and fast track—attached to the drugs in the cohort via the Drugs@FDA database [6]. A fourth designation, breakthrough therapy, was instituted in 2012, midway through the study time period, so we excluded it from the analysis. The FDA confers priority review status on therapeutics that “offer major advances in treatment, or provide a treatment where no adequate therapy exists” [7]. Accelerated approval allows for earlier marketing of agents that fill an unmet need for a serious medical condition by relying on surrogate endpoint data for initial approval [8]. The fast-track designation can be provided to similarly promising agents, allowing for closer coordination between agency and manufacturer throughout the development phase, in addition to a more streamlined review process.

We identified whether the drug had been subsequently approved for other uses, such as other subsets of the same disease or other indications. For the oncologic drugs, we searched the National Comprehensive Cancer Network (NCCN) Database for approved off-label uses. An off-label use is a use for an indication that was not part of either the original labeled indication or an additional FDA approved indication. The NCCN database is a valid proxy to ascertain the extent of off-label use, as insurance companies use it to determine coverage [9].

Finally, from the company’s U.S. Securities and Exchange Commission filings, we identified the 2014 revenues attributed to sale of the drug in the United States (and worldwide sales, if available), the most recent year of data we could consistently find. Additionally, we assessed the reported monthly cost for each drug in 2014 using the Memorial Sloan Kettering DrugAbacus drug pricing database for the oncology drugs [10] and consumer-reported databases for non-oncologic drugs [11]. We also used the DrugAbacus database to assess the prices of non-biomarker-derived oncologic orphan drugs and non-orphan oncologic drugs that were approved during the same time period (2009–2015). The dollar values reported are not specific to each clinical indication.

## Findings

From 2009 to 2015, 229 new drugs were approved, of which 84 (37%**)** had an orphan designation. The annual rate of orphan-designated drug approvals peaked in 2015, when 21/45 of the drugs in our cohort (47%) were approved with an orphan designation [1]. See S1 Appendix for full list of approved orphan drugs.

Among the 84 orphan-designated drugs, 13 (16%) were for biomarker-derived subsets of more prevalent diseases. Eleven of these addressed oncology indications. For example, afatinib (Gilotrif) was approved as an orphan drug in 2013 to treat patients with non-small cell lung cancer with an *EGFR* positive mutation, a rare variant affecting about 10% of patients [3]. Two non-oncologic drugs met inclusion criteria: ivacaftor (Kalydeco), approved in 2012 to treat the class III Gly551Asp mutation of cystic fibrosis, and lumacaftor/ivacaftor (Orkambi), approved in 2015 to treat the F508del mutation of cystic fibrosis (Table 1).

**Table 1:** Approvals of New Orphan-Designated Drugs Indicated for Biomarker-Defined Subsets of More Common Diseases, 2009–2015

Orphan-Designated Drug (Brand Name)	Approved Indication	Other Special FDA Designation	Subsequent FDA-Approved Indications	Patients Receiving Drug in Pivotal Trial(s), *n* (Phase)	Surrogate Endpoint of Pivotal Trial(s)	Estimated Cost Per Month (2014 US $ Thousand)	2014 Net Revenue from U.S. Sales (US$ Million)
**Cabozantinib (Cometriq)**	Medullary thyroid carcinoma with activating RET point mutation M918T	P, F	--	219 (Phase III)	PFS	10,229	40.1
**Ponatinib (Iclusig)**	CML with T315I mutation	A, P, F	--	449 (Phase II)	Cytogenic response	9,387	55.7
**Ivacaftor (Kalydeco)**	Cystic fibrosis mutation Gly551Asp	P, F	Y	212* (Phase III)	Improved FEV1	--**	463
**Afatinib (Gilotrif)**	EGFR mutated NSCLC (EGFR exon 19 deletions or exon 21 L858R substitution)	P, F	Y	230 (Phase III)	PFS	6,170	--
**Dabrafenib (Tafinlar)**	BRAF V600E mutated metastatic melanoma	F	Y	187 (Phase III)	PFS	9,564	87.6
**Idelalisib (Zydelig)**	CLL with p53 mutation; PI3K inhibitor	A, P, F	Y	110 (Phase III)	PFS	8,015	23
**Crizotinib (Xalkori)**	Alk+ NSCLC, Alk and ROS inhibitor	A, P, F	Y	172 (Phase III)	PFS	11,589	438
**Ceritinib (Zykadia**)	Alk+ NSCLC, specific ALK mutations	A, P	--	163 (Phase III)	Objective response rate	13,672	31
**Vemurafenib (Zelboraf)**	BRAFV600E mutated unresectable or metastatic melanoma	P, F	Y	337 (Phase III)	PFS	11,332	69.2
**Alectinib (Alecensa)**	Alk+ NSCLC, specific ALK mutations	A, P	-- †	225* (Phase III)	Objective Response Rate	--**	--‡
**Cobimetinib (Cotellic)**	BRAF V600E or V600K mutated unresectable or metastatic melanoma used with vemurafenib	P, F	-- †	247 (Phase III)	PFS	7,475	--‡
**Lumacaftor/ivacaftor (Orkambi)**	F508del mutation in cystic fibrosis	P, F	--	737 (Phase III)	Improved FEV1	--**	--‡
**Osimertinib (Tagrisso)**	EGFR T790M mutation-positive NSCLC	A, P, F	-- †	411* (Phase III)	Tumor response rate, PFS	12,735	--‡

A = Accelerated approval, P = Priority review, F = Fast track, PFS = Progression-free survival, FEV1 = Forced expiratory volume (1 second)

* Approval based on two pivotal trials

† No subsequent FDA approved indications at the time of analysis

‡ 2015 revenue data not available at time of analysis

**Cost data not present in the DrugAbacus database

Orphan drugs for biomarker-defined disease subsets represented a substantial proportion of drugs approved for oncology indications during the study time period. A total of 89 drugs were approved for oncology indications, of which 39 (44%) received orphan designation. Of the 39 orphan-designated oncology drugs, 11 (28%, or 12% of all newly approved oncology drugs) were for biomarker-derived disease subsets.

### Features of Drug Development

Three of the drugs in our sample were developed by smaller biotechnology firms that were subsequently acquired by larger pharmaceutical companies. Idelalisib (Zydelig) emerged from Calistoga Pharmaceuticals, a company focused on developing PI3K inhibitors that was later bought by Gilead Sciences [2]. Crizotinib (Xalkori) was initially attributed to Sugen, a small biotechnology firm based in California, which was later acquired by Pharmacia and subsequently Pfizer. Vemurafenib (Zelboraf) was developed in part at Plexxikon, which was later acquired by Daiichi Sankyo in 2011.

Clinical development times for this subset of orphan-designated drugs were short. All 13 drugs received at least one other expedited FDA designation besides the Orphan Drug Act designation (Accelerated Approval, Priority Review, Fast Track), with 12 receiving at least two other designations. Four of the drugs received all four designations. The median time between the initiation of human clinical trials and the submission of the full trial results dossier to the FDA was 4.6 years (interquartile range [IQR]: 3.4–5.6 years). Approvals were granted after a median FDA review period of 5.3 months.

The pivotal clinical trials for the 13 drugs in our sample had a median of 225 participants receiving active therapy, ranging from 110 (idelalisib) to 737 (lumacaftor/ivacaftor). One pivotal trial was phase II, while the others were phase III (nine of which were randomized). All trials tested surrogate endpoints, such as progression-free survival or objective response rate.

### Orphan Drug Pricing and Revenue

**Fig 2:**
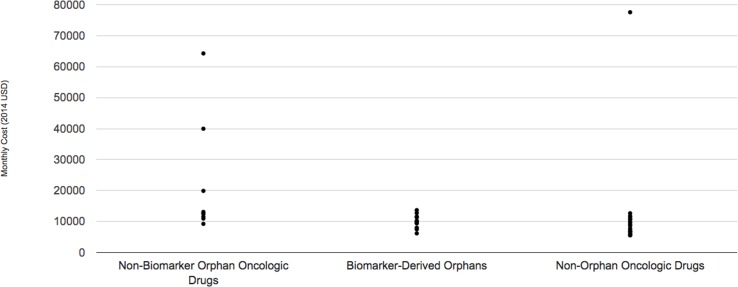
Monthly cost of three subgroups of oncologic drugs. See S1 Appendix for raw data used in these figures.

The median monthly cost for the ten biomarker-derived orphan drugs indicated for oncologic conditions included in the DrugAbacus database in 2014 dollars was US $9897, ranging from US $6,170 per month for afatinib (Gilotrif) to US $13,672 per month for ceritinib (Zykadia). By contrast, the median for non-biomarker defined oncologic orphan drugs approved during the same time period (*n* = 8) was US $12,764 per month (range: US $9,240–US $64,260). Finally, the median for non-orphan oncology drugs approved during the same time period (*n* = 19) was US $8,701 per month (range: US $5,535–US $77,554) (Fig 2).

We found 2014 U.S. sales revenues for 8 of the 9 drugs that were approved during or before 2014 (afatinib is manufactured by a European company that did not report this information). We found a wide range of revenues attributed to sales of the drug in the United States in 2014, from US $31 million (ceritinib) to US $463 million (ivacaftor). Most manufacturers did not report revenue arising from international sales, although we did observe that vemurafenib’s manufacturer reported US $302 million worldwide sales and dabrafenib’s manufacturer reported US $204 million worldwide sales in that year.

### Subsequent Use of Orphan Drugs

The majority of the drugs in our sample were used to treat additional indications after their original approvals. As of January 2016, 7 of the 14 drugs had been formally FDA-approved for at least one other use. Ivacaftor (Kalydeco) was approved in 2012 to treat a specific mutation in cystic fibrosis, but its approval has since been expanded to include eight additional cystic fibrosis mutations. Additionally, in 2015, the FDA approved the use of ivacaftor in combination with lumacaftor (Orkambi) to treat a more common cystic fibrosis mutation (F508 deletion) occurring in 70% of cystic fibrosis patients.

The eight drugs indicated for oncologic conditions and approved before 2014 had at least one off-label use listed in the NCCN database (none of the products approved in 2015 were yet listed in the database). For example, NCCN supports use of ceritinib (Zykadia), approved in 2014 for ALK+ non-small cell lung cancer, for soft tissue sarcoma and inflammatory myofibroblastic tumors with the ALK translocation. Dabrafenib (Tafinlar), approved to treat BRAF^V600E^ metastatic melanoma, can be used off label for selected subsets of central nervous system malignancies and non-small cell lung cancer.

## Discussion

We found that biomarker-defined orphan drugs, most of which relate to oncologic indications, now make up a substantial minority of orphan-designated drug approvals. This subset is characterized by short development times and high prices that are consistent with the costs of other non-orphan drugs. However, these orphan-designated drugs are also available for use outside the biomarker-defined disease subset for which they were originally approved, as nearly all of the drugs in our sample were subsequently associated with other supplemental indications.

Among the justifications for the Orphan Drug Act’s incentives are that drugs for rare diseases are costly to develop and test, and the small numbers of patients receiving them would not provide sufficient return on manufacturers’ investment. But these justifications may not apply equally to all drugs for rare diseases. In our analysis, we found that biomarker-defined orphan-designated drugs can be developed based on trials in small numbers of patients and relatively short development times. By comparison, the median clinical trial time period for non-orphan-designated cancer drugs during a similar time period was 6.9 years (IQR 6.5–8.0 years) [12]. They also sustain high prices after approval as well as broad coverage by insurers in the United States [13].

The biomarker-defined orphan-designated drugs in our sample were frequently associated with secondary approvals or guideline-supported off-label uses, although we did not assess actual off-label prescribing rates [14,15]. Is the Orphan Drug Act relevant when drugs find such additional uses after approval [16,17]? Applying the orphan designation in such circumstances wastes resources that might otherwise be applied to more deserving drugs. For example, the FDA’s US $2.17 million user fee is waived for orphan-designated drugs, and studies show that the FDA takes a highly flexible posture in its review, accepting less rigorous, smaller-scale premarket efficacy testing than for non-orphan drugs [18,19]. The fact that many drugs approved as orphan-designated products on the basis of biomarker-defined populations quickly and readily lead to additional recommended indications suggests that the FDA should reconsider whether biomarker-defined subsets of more common diseases are truly “rare diseases” in the same way as rare cancers or enzyme deficiencies, and whether they are similarly deserving of these regulatory incentives [20,21]. Scientific advances lead to the uncovering of more biomarkers, and the increasing number of biomarker-defined subsets of more common diseases that will inevitably result should lead to a re-examination of how a “rare disease” is defined in the United States to determine the applicability of the Orphan Drug Act. For example, instead of being defined based on the number of patients with a certain disease, the drug class or genomic drug target could be the basis for the designation. Thus, a drug designed to treat ALK mutations would qualify for orphan designation status if the number of patients with that mutation across all cancers, rather than just lung cancer, was less than the 200,000-patient threshold.

The prices of oncology drugs did not differ substantially whether the drug targeted a rare disease (either biomarker-defined or not) or a more common form of cancer, suggesting that pricing does not reflect either a premium for the small patient populations or a discount due to the smaller trials and generally shorter development times characterized by these products. While we do not have insight into confidential rebates that private payors may have negotiated with manufacturers, such a result suggests that drug pricing in the U.S. market is largely insensitive to development costs—which would vary based on whether the product was studied in a small or large population—and is instead more closely tied to manufacturers’ ability to set prices [22]. Recent efforts to tie oncology drug pricing closer to value may lead to changes in these trends in future years [23].

In summary, we found a substantial number of new orphan-designated drugs intended to treat biomarker-defined subsets of more common diseases. These diseases have a number of characteristics that make them ill-suited to the orphan drug designation, including rapid expansion of recommended indications after approval. Application of the Orphan Drug Act to these diseases risks wasting regulatory resources that might be better focused on truly rare conditions.

## Supporting Information

S1 Appendix Appendix lists biomarker-derived and non-biomarker-derived new orphan-designated drugs approved by the FDA, 2009–2015, as well as the data underlying Figs 1 and 2. (DOCX)
