# Supplementary material for: Biomarker-Defined Subsets of Common Diseases: Policy and Economic Implications of Orphan Drug Act Coverage
Source: PLoS Med. 2017 Jan 3;14(1):e1002190. doi: 10.1371/journal.pmed.1002190 (PMC5207392; doi:10.1371/journal.pmed.1002190)
Supplement: S1 Appendix — (DOCX) [file pmed.1002190.s001.docx]

**Appendix I: Orphan drugs approved by the FDA 2009-2015**

Biomarker-derived orphan drugs

| **Name of Drug** | Disease used to treat | Year approved |
| --- | --- | --- |
| **Cabozantinib (Cometriq)** | Medullary Thyroid Carcinoma with activating RET point mutation M918T | 2012 |
| **Ponatinib (Iclusig)** | CML with T315I mutation | 2012 |
| **Ivacaftor (Kalydeco)** | Cystic Fibrosis mutation Gly551Asp | 2012 |
| **Afatinib (Gilotrif)** | EGFR mutated NSCLC (EGFR exon 19 deletions or exon 21 L858R substitution) | 2013 |
| **Dabrafenib (Tafinlar)** | BRAF V600E mutated metastatic melanoma | 2013 |
| **Idelalisib (Zydelig)** | CLL with p53 mutation; PI3K inhibitor | 2014 |
| **Crizotinib (Xalkori)** | Alk+ NSCLC, Alk and ROS inhibitor | 2011 |
| **Ceritinib (Zykadia**) | Alk+ NSCLC, specific ALK mutations | 2014 |
| **Vemurafenib (Zelboraf)** | BRAF mutated melanoma | 2011 |
| **Alectinib (Alecensa)** | Alk+ NSCLC, specific ALK mutations | 2015 |
| **Cobimetinib (Cotellic)** | BRAF mutated melanoma, used with vemurafefnib | 2015 |
| **Lumacaftor/ivacaftor (Orkambi)** | F508del mutation in cystic fibrosis | 2015 |
| **Osimertinib (Tagrisso)** | EGFR T790M mutation-positive NSCLC | 2015 |

Non-biomarker derived orphan drugs

| **Isavuconazonium sulfate (Cresemba)** | Antifungal for invasive aspergillosis and mucormycosis | 2015 |
| --- | --- | --- |
| **Necitumumab (Portrazza)** | EGFR antagonist for NSCLC | 2015 |
| **Idarucizumab (Praxbind)** | Reversal agent for dabigatran | 2015 |
| **Evolocumab (Repatha)** | monoclonal antibody to PCSK9 to treat hyperlipidemia | 2015 |
| **Dinutuximab (Unituxin)** | Chimeric monoclonal antibody for neuroblastoma | 2015 |
| **Selexipag (Uptravi)** | Prostacyclin receptor agonist to treat pulmonary arterial hypertension | 2015 |
| **Daratumumab (Darzalex)** | anti-CD38 monoclonal antibody for Multiple Myeloma | 2015 |
| **Elotuzumab (Empliciti)** | Immunostimulatory antibody for Multiple Myeloma | 2015 |
| **Panobinostat (Farydak)** | Histone deacetylase inhibitor to treat Multiple Myeloma | 2015 |
| **lenvatinib (Lenvima)** | Tyrosine kinase inhibitor to treat differentiated thyroid cancer | 2015 |
| **Parathyroid hormone (Natpara)** | injectable PTH to treat hypoparathyroidism | 2015 |
| **Ixazomib (Ninlaro)** | proteasome inhibitor to treat multiple myeloma | 2015 |
| **Yondelis (Trabectedin)** | Transcription inhibitor to treat soft tissue sarcoma | 2015 |
| **Bosutinib monohydrate (Bosulif)** | Bcr-Abl Kinase inhibitor for imatinib resistant CML | 2012 |
| **Carfilzomib (Kyprolis)** | selective proteasome inhibitor to treat multiple myeloma | 2012 |
| **Omacetaxine mepesuccinate (Synribo)** | Tyrosine kinase inhibitor to treat CML | 2012 |
| **Obinutuzumab (Gazyva)** | Monoclonal antibody to treat CLL | 2013 |
| **Pomalidomide (Pomalyst)** | Immunomodulator used to treat multiple myeloma | 2013 |
| **Belinostat (Beleodaq)** | Histone deacetylase inhibitor to treat peripheral T cell lymphoma | 2014 |
| **Blinatumomab (Blincyto)** | Monoclonal antibodies for B-cell ALL | 2014 |
| **Ramucirumab (Cyramza)** | Monoclonal antibody to VEGFR2 used to treat NSCLC | 2014 |
| **Nivolumab (Opdivo)** | Anti-PD1 monoclonal antibody to treat metastatic melanoma | 2014 |
| **Siltuximab (Sylvant)** | anti-IL6 chimeric monoclonal antibody for Castleman’s disease | 2014 |
| **Ofatumumab (Arzerra)** | Monoclonal antibody used to treat CLL | 2009 |
| **Pralatrexate (Folotyn)** | Antifolate used to treat peripheral T cell lymphoma | 2009 |
| **Romidepsin (Istodax)** | Histone deacetylase inhibitor  peripheral T cell lymphoma | 2009 |
| **Brentuximab Vedotin (Adcetris)** | Antibody-drug conjugate to treat Hodgkin’s Lymphoma and Anaplastic Large Cell Lymphoma | 2011 |
| **Vandetanib (Caprelsa)** | Kinase inhibitor to treat medullary thyroid cancer | 2011 |
| **Ruxolitinib (Jakafi)** | JAK inhibitor to treat myelofibrosis | 2011 |
| **Ipilimumab (Yervoy)** | monoclonal Ig to treat metastatic melanoma | 2011 |
| **Nintedanib (Ofev)** | TK inhibitor to treat idiopathic pulmonary fibrosis | 2011 |
| **Bedaquiline fumarate (Sirturo)** | ATP synthase inhibitor to treat multidrug resistant TB | 2012 |
| **Raxibacumab** | Monoclonal antibody used to treat inhaled anthrax | 2012 |
| **Macitentan (Opsumit)** | Endothelin receptor antagonist for Pulmonary artery hypertension | 2013 |
| **Ibrutinib (Imbruvica)** | Inhibitor of Bruton tyrosine kinase to treat Mantle cell lymphoma | 2013 |
| **Pirfenidone (Esbriet)** | Downregulates production of GF to treat idiopathic pulmonary fibrosis | 2014 |
| **Tasimelteon (Hetlioz)** | selective agonist for melatonin receptors for non-24 sleep wake disorder | 2014 |
| **Miltefosine (Impavido)** | Akt inhibtor used in Leishmaniasis | 2014 |
| **Olaparib (Lynparza)** | PARP inhibitor for germline BRCA mutations in ovarian cancer | 2014 |
| **Droxidopa (Northera)** | Prodrug to norepinephrine and epinephrine used in neurogenic orthostatic hypotension | 2014 |
| **Belatacept (Nulojix)** | Fusion protein for acute rejection of kidney transplant | 2011 |
| **Glucarpidase (Voraxaze)** | Recombinant enzyme used for methotrexate toxicity | 2012 |
| **Pembrolizumab (Keytruda)** | Metastatic melanoma; targets PD-1 receptor | 2014 |
| **Taliglucerase alfa (Elelyso)** | Recombinant glucocerebrosidase to treat Gaucher disease | 2012 |
| **Teduglutide (Gattex)** | GLP-2 analog to treat short bowel syndrome | 2012 |
| **Lomitapide (Juxtapid)** | Inhibits MTTP, treats homozygous familial hypercholesterolemia | 2012 |
| **Pasireotide diaspartate (Signifor)** | Somatostatin analog to treat Cushing’s disease | 2012 |
| **Riociguat (Adempas)** | Stimulates sGC to treat chronic thromboembolic pulmonary hypertension | 2013 |
| **Trametinib (Mekinist)** | Inhibits MEK1 and 2 to treat metastatic melanoma | 2013 |
| **Mipomersen sodium (Kynamro)** | Targets mRNA for apoB homozygous familial hypercholesterolemia | 2013 |
| **Eliglustat (Cerdelga)** | glucosylceramide synthase inhibitor to treat Gaucher disease | 2014 |
| **Metreleptin (Myalept)** | Synthetic analog of leptin to treat generalized lipodystrophy | 2014 |
| **Elosulfase alfa (Vimizim)** | Enzyme replacement for  Morquio syndrome | 2014 |
| **Clobazam (Onfi)** | Benzodiazepine for  Lennox-Gastaut syndrome | 2011 |
| **Cholic acid (Cholbam)** | enzyme replacement for bile acid synthesis disorders, Zellweger spectrum disorders | 2015 |
| **Sebelipase alfa (Kanuma)** | Recombinant human LAL for  Lysosomal Acid Lipase deficiency | 2015 |
| **Strensiq (asfotase alfa)** | Enzyme replacement therapy for perinatal / infantile and juvenile-onset hypophosphatasia (HPP) | 2015 |
| **Uridine triacetate (Xuriden)** | Pyrimidine analog used to treat Hereditary Orotic Aciduria | 2015 |
| **Artemether / Lumefantrine (Coartem)** | Used to treat malaria that is resistant to chloroquine | 2009 |
| **Canakinumab (Ilaris)** | Targets IL-1 beta to treat cryopyrin-associated periodic syndromes | 2009 |
| **Ecallantide (Kalbitor)** | Inhibits protein kallikrein to treat hereditary angioedema | 2009 |
| **Vigabatrin (Sabril)** | Inhibits GABA transaminase to treat epilepsy | 2009 |
| **Dalfampridine (Ampyra)** | 4-aminopyridine to treat multiple sclerosis | 2010 |
| **Carglumic acid (Carbaglu)** | Treat hyperammonemia in N-acetylglutamate synthase deficiency | 2010 |
| **Pegloticase (Krystexxa)** | Recombinant uricase used to treat severe, chronic gout | 2010 |
| **Alglucosidase alfa (Lumizyme)** | Enzyme replacement therapy for Pompe disease | 2010 |
| **Velaglucerase alfa (Vpriv)** | Recombinant glucoerebrosidase for long term replacement therapy for Gaucher disease | 2010 |
| **Collagenase clostridium histolyticum** | Enzyme that dismantles collagen used to treat Dupuytren’s contracture | 2010 |
| **Asparaginase Erwinia chrysanthemi (Erwinaze)** | Treats patients with ALL who have developed allergy to certain chemotherapeutic agents | 2011 |
| **Icatibant acetate (Firazryr)** | Peptidomimetic drug used to treat hereditary angioedema caused by C1-esterase inhibitor deficiency | 2011 |
| **Deferiprone (Ferriprox)** | Iron chelating agent to treat iron toxicity | 2011 |

**Appendix II: Data for Figure 1: Orphan Drug Act designations by year**

| Year | Number of drugs receiving Orphan Drug Designation |
| --- | --- |
| 2000 | 70 |
| 2001 | 78 |
| 2002 | 64 |
| 2003 | 95 |
| 2004 | 132 |
| 2005 | 123 |
| 2006 | 142 |
| 2007 | 119 |
| 2008 | 165 |
| 2009 | 164 |
| 2010 | 195 |
| 2011 | 203 |
| 2012 | 190 |
| 2013 | 261 |
| 2014 | 291 |
| 2015 | 353 |

**Appendix III:** **Data for Figure 2:** **2014 monthly cost of three subgroups of oncologic drugs**

| Non-biomarker Orphan drugs for oncologic condition |  |
| --- | --- |
| Blinatumomab (Blincyto) | $64260 |
| Ipilimumab (Yervoy) | 39947 |
| Nivolumab (Opdivo) | 12435 |
| Ofatumumab (Arzerra) | 19884 |
| Pembrolizumab (Keytruda) | 9240 |
| Pomalidomide (Pomalyst) | 11520 |
| Ramucirumab (Cyramza) | 13093 |
| Vandetanib (Caprelsa) | 10959 |

| Biomarker-Derived Orphan Drugs |  |
| --- | --- |
| Afatinib (Gilotrif) | $6,170 |
| Cabozantinib (Cometriq) | 10,229 |
| Ceritinib (Zykadia) | 13,672 |
| Cobimetinib (Cotellic) | 7475 |
| Crizotinib (Xalkori) | 11,589 |
| Dabrafenib (Tafinlar) | 9,564 |
| Idelalisib (Zydelig) | 8,015 |
| Osimertinib (Tagrisso) | 12735 |
| Ponatinib (Iclusig) | 9387 |
| Vemurafenib (Zelboraf) | 11,332 |

| Non-orphan Oncologic Drugs |  |
| --- | --- |
| Abiraterone (Zytiga) | $5535 |
| Ado-trastuzumab emtansine (Kadcyla) | 10807 |
| Axitinib (Inlyta) | 9580 |
| Bevacizumab (Avastin | 5551 |
| Cabazitaxel (Jevtana) | 11771 |
| Enzalutamide (Xtandi) | 7298 |
| Eribulin (Halaven) | 6724 |
| Everolimus (Afinitor) | 8701 |
| Obinutuzumab (Gazyva) | 5878 |
| Paclitaxel protein-bound (Abraxane) | 6837 |
| Palbociclib (Ibrance) | 10690 |
| Pazopanib (Votrient) | 6635 |
| Pertuzumab (Perjeta) | 7783 |
| Radium Ra 223 dichloride (Xofigo) | 12657 |
| Regorafenib (Stivarga) | 9919 |
| Sipuleucel-T (Provenge) | 77554 |
| Sorafenib (Nexavar) | 6178 |
| Trametinib (Mekinist) | 8955 |
| Ziv-aflibercept (Zaltrap) | 11407 |

|  |
| --- |
